# Resistome and Phylogenomics of *Escherichia coli* Strains Obtained from Diverse Sources in Jimma, Ethiopia

**DOI:** 10.3390/antibiotics14070706

**Published:** 2025-07-14

**Authors:** Mulatu Gashaw, Esayas Kebede Gudina, Guenter Froeschl, Ralph Matar, Solomon Ali, Liegl Gabriele, Amelie Hohensee, Thomas Seeholzer, Arne Kroidl, Andreas Wieser

**Affiliations:** 1 School of Medical Laboratory Science, Jimma University, Jimma P.O. Box 378, Ethiopia; 2 CIH^LMU^ Center for International Health, University Hospital, LMU Munich, Leopoldstrasse 5, 80802 Munich, Germany; 3 Department of Medicine, Jimma University, Jimma P.O. Box 378, Ethiopia; 4 Institute of Infectious Diseases and Tropical Medicine, University Hospital, Ludwig-Maximilians-Universität, 80802 Munich, Germany; 5 Fraunhofer Institute for Translational Medicine and Pharmacology ITMP, Immunology, Infection and Pandemic Research IIP, Türkenstraße 87, 80799 Munich, Germany; 6 Department of Microbiology, Parasitology, and Immunology, St. Paul’s Hospital Millennium Medical College, Addis, Ababa P.O. Box 1176, Ethiopia; 7 Max von Pettenkofer-Institute (Medical Microbiology), LMU Munich, 80336 Munich, Germany; 8 German Center for Infection Research (DZIF), Partner Site Munich, 80802 Munich, Germany

**Keywords:** phylogeny, resistome, antimicrobial resistance, *E. coli*, One Health

## Abstract

**Introduction**: In recent years, antimicrobial resistance (AMR) rates have increased significantly in bacterial pathogens, particularly extended beta-lactam resistance. This study aimed to investigate resistome and phylogenomics of *Escherichia coli* (*E. coli*) strains isolated from various sources in Jimma, Ethiopia. **Methods**: Phenotypic antibiotic resistance patterns of *E. coli* isolates were determined using automated Kirby–Bauer disc diffusion and minimum inhibitory concentration (MIC). Isolates exhibiting phenotypic resistance to beta-lactam antibiotics were further analyzed with a DNA microarray to confirm the presence of resistance-encoding genes. Additionally, multilocus sequence typing (MLST) of seven housekeeping genes was conducted using PCR and Oxford Nanopore-Technology (ONT) to assess the phylogenetic relationships among the *E. coli* isolates. **Results**: A total of 611 *E. coli* isolates from human, animal, and environmental sources were analyzed. Of these, 41.6% (254) showed phenotypic resistance to at least one of the tested beta-lactams, 96.1% (244) thereof were confirmed genotypically. More than half of the isolates (53.3%) had two or more resistance genes present. The most frequent ESBL-encoding gene was CTX-M-15 (74.2%; 181), followed by TEM (59.4%; 145) and CTX-M-9 (4.1%; 10). The predominant carbapenemase gene was NDM-1, detected in 80% (12 out of 15) of carbapenem-resistant isolates. A phylogenetic analysis revealed clonality among the strains obtained from various sources, with international high-risk clones such as ST131, ST648, ST38, ST73, and ST405 identified across various niches. **Conclusions**: The high prevalence of CTX-M-15 and NDM-1 in multidrug-resistant *E. coli* isolates indicates the growing threat of AMR in Ethiopia. The discovery of these high-risk clones in various niches shows possible routes of transmission and highlights the necessity of a One Health approach to intervention and surveillance. Strengthening antimicrobial stewardship, infection prevention, and control measures are crucial to mitigate the spread of these resistant strains.

## 1. Introduction

*Escherichia coli (E. coli)* is the most frequently isolated and studied bacteria in microbiology [1]. Although it is naturally present in human gut microbiota, factors such as frequent contact with animals and their manures, inadequate food hygiene, and poor infection prevention practices might expose people even more to this pathogen [2,3]. This exposure can lead to food and water borne diseases, as well as healthcare-associated and community-acquired infections in humans, with relatively few infections occurring in animals [4,5,6,7,8]. In recent years, the rise in AMR in *E. coli* has become a major challenge in public health, and it is often considered as a surrogate marker of antibiotic resistance in all *Enterobacterales* [9,10].

*E. coli* exhibits resistance to a wide range of antibiotics, increasingly to penicillins, cephalosporins, and carbapenems, primarily due to extended-spectrum beta-lactamase (ESBL) and/or carbapenemase production [11]. ESBLs are a group of highly mobile antibiotic resistance genes that confer resistance against certain beta-lactam antibiotics by hydrolyzing penicillins, monobactams, and third- and fourth-generation cephalosporins, but not cephamycins or carbapenems [12]. Infections due to ESBL-producing organisms are significantly more difficult to treat. Thirty years ago, the most commonly identified ESBL genes were TEM or SHV variants. However, CTX-M-type enzymes have now become the most prevalent, with the CTX-M-15 variant being the most widespread globally, followed by CTX-M-14 and CTX-M-27 [11,12]. Additionally, certain carbapenemase-encoding genes, including KPC, OXA-48, IMP, VIM, and NDM, can hydrolyze nearly all β-lactams, leading to resistance to multiple antibiotic classes and severely limiting treatment options, even more so than ESBLs [13].

According to previous studies, the presence of a high rate of ESBL- and carbapenem-resistant *E. coli* strains in animals and the environment suggests that these serve as a common reservoir for these bacteria. Particularly, the gut flora, sewage, and soil are typical reservoirs. The accumulation and spread of resistant *E. coli* variants highlights the urgent need for effective AMR management strategies, especially considering the challenges associated with developing new antimicrobial agents [14].

Many reports on AMR primarily refer to clinical data, while there is limited information regarding environmental and animal samples as reservoirs and potential transmission routes for AMR between the different reservoirs [2,15,16]. This highlights the growing need for a One Health approach to address the problems related to AMR, and helps to understand the nexus between humans, animals, and the natural environment [16]. Therefore, this study aimed to investigate the amount of ESBL- and/or carbapenem-resistant *E. coli* strains in clinical, animal, and environmental samples in Jimma Town, Ethiopia, as well as to explore their phylogenetic similarities across these various sources.

## 2. Results

### 2.1. Phenotypic Antimicrobial Susceptibility Test Results

**Figure 1:**
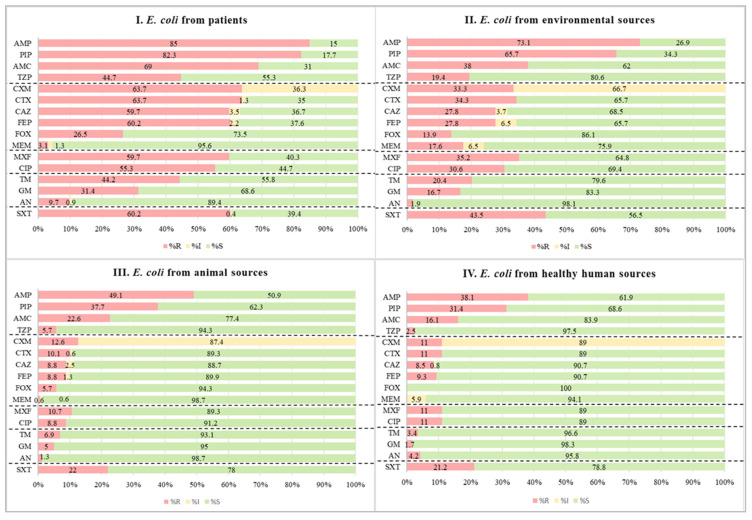
Analysis of antimicrobial resistance patterns of *E. coli* across four sources: patients (**I**), environmental samples (**II**), animals (**III**), and healthy individuals (**IV**). Key for the tested antibiotics: AMP, ampicillin; AMC, amoxicillin/clavulanic acid; PIP, piperacillin; TZP, piperacillin-tazobactam; CAZ, ceftazidime; CXM, cefuroxime; CTX, cefotaxime; ceftazidime; FEP, cefepime; FOX, cefoxitin; MEM, meropenem; MXF, moxifloxacin; CIP, ciprofloxacin; GM, gentamicin; TM, tobramycin; AN, amikacin; and SXT, sulfamethoxazole-trimethoprim. Red: proportion of resistant (R) isolates; yellow: proportion of intermediate/increased exposure (I) isolates; green: proportion of susceptible (S) isolates.

A total of 611 *E. coli* isolates were collected from various sources in Jimma, Ethiopia, to investigate antimicrobial resistance patterns and genetic diversity. Of these, 226 were from patients (obtained from various clinical samples, including blood, cerebrospinal fluid [CSF], wound swabs, ascitic fluid, pleural fluid, abscesses, peritoneal fluid, and synovial fluid), 118 from healthy individuals (feces), 159 from animals (feces, rectal swabs, or droppings), and 108 from environmental sources (surface swabs, sewage, or flies). Phenotypic antimicrobial susceptibility testing (AST) was performed using a panel of commonly prescribed antibiotics, including cefuroxime, ampicillin, piperacillin, meropenem, and amikacin. Notably, none of the *E. coli* isolates were susceptible to cefuroxime. In contrast, resistance was observed in 64.6% of isolates for ampicillin and 58.1% for piperacillin, while resistance rates remained low for meropenem (5.9%) and amikacin (5.1%) (Figure 1). When comparing resistance rates among *E. coli* strains from different sources, clinical isolates exhibited the highest resistance to ampicillin (85.0%) and piperacillin (82.3%), while animal isolates had lower resistance rates at 49.1% and 37.7%, respectively (Figure 1). Furthermore, 56.5% of *E. coli* strains were identified as MDR, following the criteria set by Magiorakos et al., 2012 [17]. Notably, the highest proportion of MDR isolates (76.1%) was observed among clinical isolates.

### 2.2. Minimal Inhibitory Concentrations (MICs) for Carbapenem-Resistant Isolates

**Figure 2:**
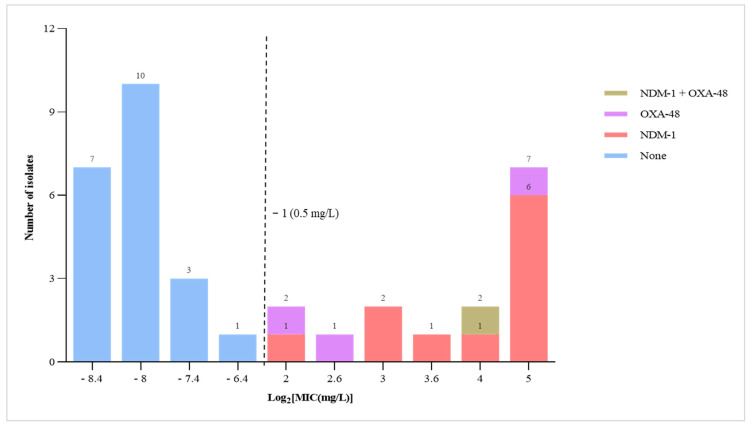
Minimum inhibition concentration of ertapenem for *E. coli* isolates. Note: 0.5 mg/L is the clinical break point for ertapenem according to EUCAST guidelines for MICs.

The MICs of carbapenem-resistant *E. coli* strains were measured using the E-test^®^ ertapenem strip, which is the recommended carbapenem antibiotic to screen for carbapenem resistance in Enterobacterales. In phenotypic AST results, 5.9% (36/611) of *E. coli* isolates exhibited some level of resistance to meropenem. Of these, 41.7% (15) were above the clinical breakpoints of ertapenem (MIC > 0.5 mg/L), which is interpreted as resistant in accordance with the EUCAST guidelines [18]. Furthermore, all *E. coli* isolates with an ertapenem MIC of ≥4 mg/L were later confirmed to carry carbapenemase-encoding genes (e.g., NDM, OXA-48), as detailed in the following section (Figure 2).

### 2.3. Distribution and Co-Occurrence of β-Lactamase and Carbapenemase-Encoding Genes

In the phenotypic AST, 41.6% (254/611) of *E. coli* isolates exhibited resistance to at least one of the tested β-lactam antibiotics. Of these, 96.1% (244/254) demonstrated consistent genotypic matches with their phenotypic susceptibility results. However, the phenotypic resistance of the remaining *E. coli* strains (3.9%; 10) could not be explained by any of the identified genes, indicating the possibility of other mechanisms of resistance. Regarding the distribution of isolates with confirmed β-lactamase resistance genes, a high proportion (66.8%; 151/226) originated from patient specimens followed by environmental samples, which accounted for 54.6% (59/108) (Table 1).

Among the β-lactamase genes detected by the DNA microarray kit, our study revealed four different subtypes of β-lactamase genes, including CTX-M-9, CTX-M-15, CTX-M-32, and an undetermined CTX-M, ND enzyme, in 39.9% (244/611) of the isolates. Of these, CTX-M-15 was the predominant subtype, detected in 29.6% (181/611) of *E. coli* strains, with 20.4% (125/611) from patient isolates and 10.6% (33/611) from environmental samples. The next most common subtype was TEM (WT), accounting for 23.6% (144/611) of the isolates, with 14.6% (89/611) from patients and 5.9% (36/611) from the environment (Table 1). Additionally, 2.5% (15/611) of *E. coli* strains exhibited carbapenemase-encoding genes (NDM and OXA-48) among the tested genes (KPC, NDM, OXA-48, VIM, IMP, GES, GIM, SPM, OXA-23, OXA-24, and OXA-58). Notably, *E. coli* strains obtained from environmental sources had a higher proportion of carbapenemase-encoding genes, at 5.6% (6/108) compared to 3.5% (8/226) from patient sources (Table 1).

The most frequently coexisting β-lactamase genes were CTX-M-15 and TEM (WT), detected in 34.8% (85/244) of the strains. Additionally, two isolates harbored four different encoding genes: one with NDM + CMY II + CTX-M-15 + TEM and another with OXA-48 + NDM + CTX-M-15 + TEM. Furthermore, 8.2% (20/244) of the strains contained three encoding genes, while 44.3% (108/244) had two (Table 2). As expected, *E. coli* strains in this study exhibited a high prevalence of phenotypic resistance to penicillins and cephalosporins.

### 2.4. Multilocus Sequence Type (MLST) Comparison

**Figure 3:**
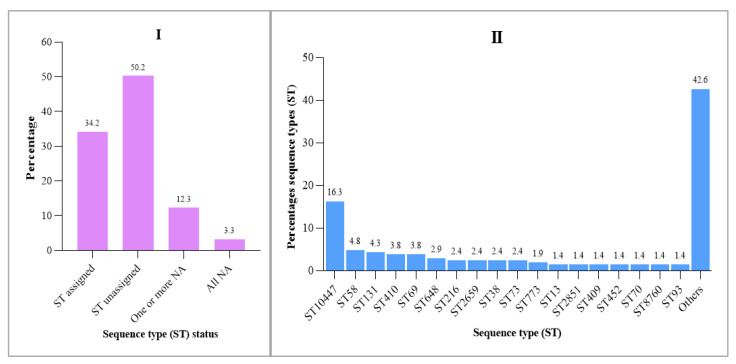
Distribution of *E. coli* strains with assigned sequence types and their frequencies. Key: “**One or More NA**” refers to *E. coli* isolates in which at least one of the housekeeping genes had no sequence reads; “**All NA**” refers to *E. coli* isolates where none of the seven housekeeping genes had any sequence reads.

Out of the 611 *E. coli* strains analyzed, successful traditional MLST comparisons were carried out for 84.4% (*n* = 516) of the strains that were obtained from various sources. This is because 12.3% (75) did not yield successful sequences for one or more of the seven housekeeping genes examined. Additionally, 3.3% (20) of the *E. coli* strains failed to produce successful sequences for all seven housekeeping genes. Interestingly, we were only able to assign sequence types (STs) to 34.2% (209) of the strains (Figure 3I), the rest comprised strains not yet represented in the database. Among the assigned strains, ST10447 was the most frequently identified sequence type, followed by ST58, ST131, ST410, ST69, ST648, and many others (Figure 3II).

**Figure 4:**
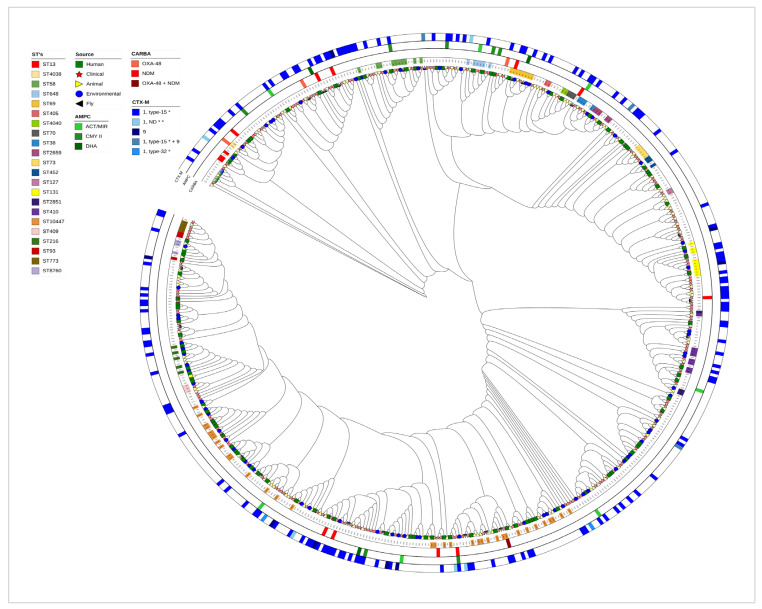
Phylogenetic tree of *E. coli* isolated from various sources and their AMR-encoding genes. The tree was constructed using the consensus sequence derived from seven housekeeping genes of *E. coli*. Different colors in the left box indicate the sequence type, AMR-encoding genes, and the origins of the *E. coli* strains. Similar sequence types are highlighted with corresponding colors.

The phylogenomic analysis of *E. coli* strains, based on sequences from the seven housekeeping genes (adk, fumC, icd, purA, gyrB, recA, and mdh), revealed distinct clades that illustrate their genetic relationships, with varying degrees of similarity. This analysis showed evidence of bacterial transmission among different clades, including resistant strains. The most frequently observed sequence types (STs) associated with ESBL were ST69 (5), ST131 (5), ST648 (4), ST10447 (4), ST38 (3), ST410 (3), and ST2659 (3). Moreover, multiple international high-risk clones, such as ST131, ST648, ST38, ST73, ST405, and ST1193, were identified across various niches (Figure 4). These STs were identified across multiple clades and sources, suggesting that resistant strains are not limited to hospitals and their surrounding areas, but are already disseminated throughout the ecosystem, including in animals, the environment, and the microbiomes of apparently healthy humans.

### 2.5. Phylogenetic Evidence of E. coli Transmission Across Human and Environmental Reservoirs

**Figure 5:**
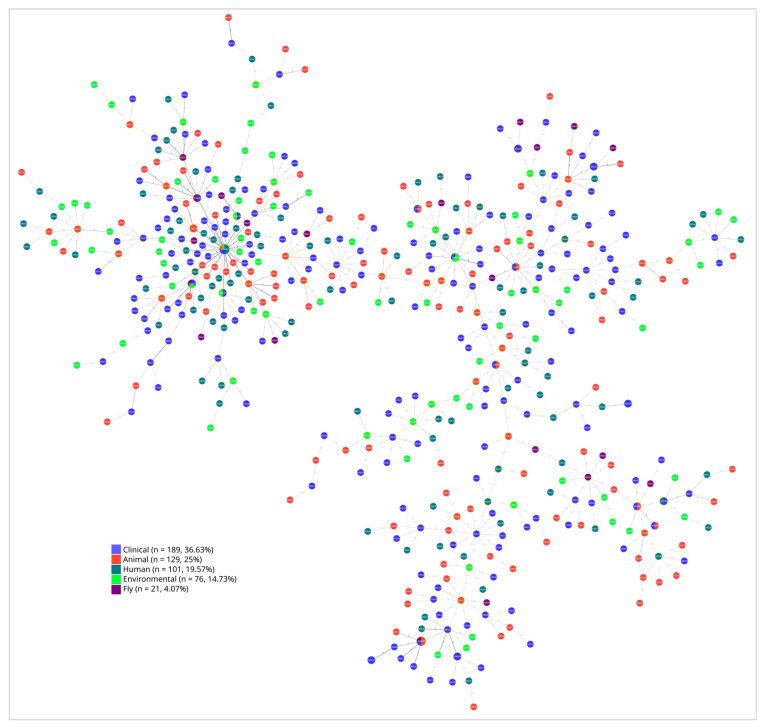
goeBURST analysis of *E. coli* strains obtained from various sources. Each dot represents an isolate from a specific source, with colors indicating the source of each isolate. Isolates were regarded as closely related or similar based on the distance between them. The shortest distances between the dots suggest potential transmission.

The constructed family tree of all successfully analyzed *E. coli* strains provides insights into the transmission dynamics and key hotspots within the study area. For instance, the large cluster in the figure suggests that *E. coli* isolates from patients, along with those from sewage and fly samples collected from the hospital and its surroundings, may have been transmitted to other sources. It also suggests interspecies transmission pathways, underscoring the importance of animal, fly, and environmental reservoirs of *E. coli*. Furthermore, the findings show the interconnectedness of humans, animals, and the environment and their potential roles in facilitating the dissemination of bacteria, including resistant strains (Figure 5).

## 3. Discussion

*E. coli* serves as a prototype bacterium to monitor the magnitude and spread of AMR-encoding genes within the Enterobacterales family. Our study provides information on the epidemiology, AMR, and phylogenetic relationships of *E. coli* strains isolated from different sources in Jimma. The study revealed a high phenotypic resistance rate among *E. coli* strains against the most frequently prescribed antibiotics in healthcare settings (Figure 1). This is supported by the identification of β-lactam-encoding genes (ESBL, AmpC, TEM, SHV, and carbapenemases) in a large proportion of the strains (Table 1). Similar to our findings, previous studies conducted in comparable settings reported resistance rates of 88.4% for cefuroxime and 81.0% for ampicillin [19,20]. Additionally, studies from other African countries reported that 89%, 84%, and 80% of the isolates were resistant to cefuroxime, ampicillin, and piperacillin, respectively [21]. In our current study, we found even higher rates of phenotypic resistance among clinical samples, with 100% resistance to cefuroxime, 85% to ampicillin, and 82.3% to piperacillin. These findings highlight the pressing issue of AMR and its implications for public health.

In this study, 33.4% (204) of *E. coli* strains were carrying CTX-M enzymes, predominantly CTX-M-15 (29.6%; 181), followed by CTX-M-9 (1.5%; 9), CTX-M-32 (0.5%; 3), and other groups under CTX-M group-1 (1.3%; 8) (Table 1). Consistent with this report, CTX-M-15 was the predominant CTX-M subtype among *E. coli* strains in previous studies performed in Jimma, Ethiopia, as well as other low- and middle-income countries in Africa [22,23,24,25,26]. However, unlike previous studies in Ethiopia, we found new CTX-M β-lactam-encoding genes from the CTX-M-1 group, particularly CTX-M-32 and other subgroups within CTX-M group-1 (Table 1). The details of these other groups were not specified further. The high prevalence of these beta-lactamases leads to increased resistance to first-line antibiotics. Therefore, continuous epidemiological monitoring and susceptibility testing of clinical isolates are advisable to ensure effective treatment, especially in resource-limited countries like Ethiopia, where antibiotic options remain limited.

Our research also revealed two genes that are responsible for carbapenemase production in *E. coli* strains phenotypically resistant to meropenem and/or ertapenem. We detected NDM-1 in 1.8% (11/611) of the isolates and OXA-48 in 0.5% (3/611). Additionally, one *E. coli* strain carried both NDM-1 and OXA-48, contributing to its resistance to the tested carbapenem antibiotics (Table 1). Most of the carbapenem-resistant *E. coli* strains were isolated from patient and environmental samples, and many of these strains contained the NDM gene, which is consistent with previous studies conducted globally [27,28]. However, five years ago, this gene was only detected in Acinetobacter baumannii strains isolated from clinical samples in the same study area [29]. This change may have resulted from the horizontal transfer of resistance genes between species [30,31]. Consequently, materials containing these resistant strains could serve as reservoirs for the further dissemination of AMR. Collaboration among healthcare professionals, veterinarians, and policymakers under the One Health framework is crucial to mitigate the spread of such multidrug-resistant pathogens.

Consistent with our findings, the co-existence of multiple types of carbapenemase-, AmpC-, and ESBL-encoding genes within the same strain has been frequently reported in previous studies from several parts of the globe [32,33]. Most importantly, our study identified two *E. coli* isolates that simultaneously harbored four different genes associated with ESBL, AmpC, and carbapenemases. In addition, the other 128 isolates harbored two or three β-lactam-encoding genes, predominantly the CTX-M and TEM (WT) types (Table 2). This high rate of co-expression of resistance genes has also been observed in previous studies conducted in Ethiopia and other sub-Saharan African countries [21,22,23].

The observed genetic similarity among *E. coli* strains obtained from various sources, including patients, animals, sewage, and environmental samples, raises significant concerns regarding the spread of AMR (Figure 3 and Figure 4). The presence of these resistant strains among various sources can create a complex web of dissemination and infection risk [34]. This interconnectedness suggests that AMR is not confined to clinical settings but can emerge and proliferate in community and environmental contexts as well [35]. The potential for resistant strains to circulate among these different sources complicates treatment options and heightens the urgency for a One Health approach for surveillance and intervention.

Additionally, identifying clusters or hotspots of transmission is crucial for infection prevention and control, particularly concerning resistant bacteria [36]. By locating areas with high rates of resistant bacteria, stakeholders can implement targeted interventions, such as enhanced surveillance and improved hygiene practices, to mitigate its spread and contain outbreaks [34]. Furthermore, understanding the dynamics of these clusters can inform stakeholders about the transmission pathways of *E. coli*, ultimately leading to more effective containment strategies and better health outcomes for the community.

### Strengths and Limitations of the Study

The strength of this study is its comprehensive analysis of a large number of *E. coli* strains from various sources, which provides valuable insights into the resistome and phylogenomic diversity in the study area, Jimma, Ethiopia. This approach enhances our understanding of antibiotic resistance patterns and their potential public health implications. However, there are some limitations to consider: (1) Not all *E. coli* strains could be assigned sequence types, as only specific housekeeping genes were used for sequencing and phylogenetic analysis and if the particular sequences were not part of the database, they could not be assigned. (2) Since this was a single-site study, the strains may not fully represent the broader population of *E. coli* in Ethiopia. These points should be considered during the interpretation of the results.

## 4. Materials and Methods

### 4.1. Study Settings and Sample Collections

**Figure 6:**
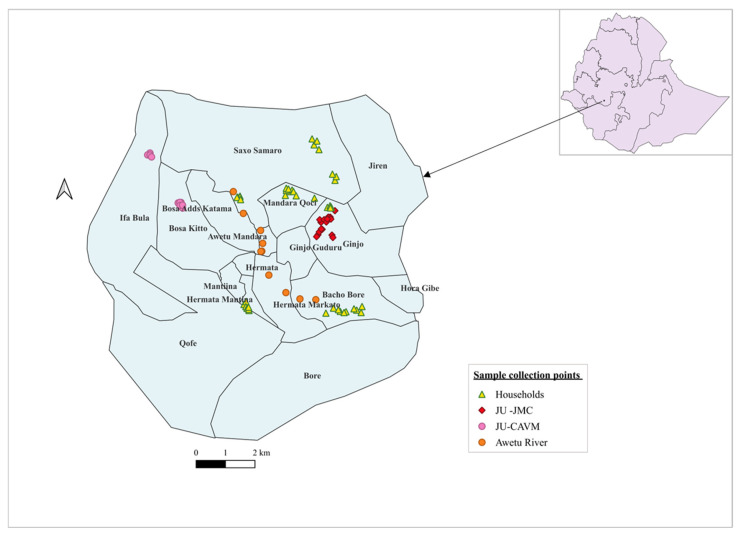
Map of Jimma town showing the locations where various samples were collected. Key: JU-JMC, Jimm University Jimma Medical Center; JU-CAVM, Jimma University College of Agriculture and Veterinary Medicine.

A cross-sectional study was conducted at Jimma Medical Center (JMC) and in Jimma town from 2019 to 2021. Samples collected for analysis included feces from healthy humans, rectal swabs, and droppings/feces from animals at various households in Jimma town. Additionally, surface swabs from hospital buildings and medical devices, along with sewage and flies from the hospital and surrounding areas, as well as various clinical samples from patients at the hospital, were collected (Figure 6). These samples were collected by clinicians, nurses, patients, or environmental health professionals, and were processed following the standard operating procedures for each type of specimen.

### 4.2. Culture and Identification

After the samples were collected and transported to the microbiology laboratory, they were inoculated onto MacConkey or blood agars (Oxoid, Cambridge, UK) and incubated aerobically at 35 °C for 18 to 22 h. Following identification through a series of biochemical tests, the purified isolates were grown on nutrient agar (Oxoid, Cambridge, UK) and stored at −80 °C in storage media. Moreover, all strains were re-identified at the Max von Pettenkofer Institute using matrix-assisted laser desorption ionization–time of flight (MALDI-TOF) mass spectrometry (Bruker, Bremen, Germany).

### 4.3. Qualitative Phenotypic Antimicrobial Susceptibility Tests

The antimicrobial susceptibility test was carried out using a semi-automated Kirby–Bauer disc diffusion technique against ampicillin (10 μg), amoxicillin/clavulanic acid (10 μg), piperacillin (30 μg), piperacillin/tazobactam (30 μg), cefuroxime (30 μg), ceftazidime (30 μg), cefotaxime (30 μg), cefoxitin (30 μg), cefepime (30 μg), meropenem (10 μg), gentamicin (10 μg), tobramycin (10 μg), amikacin (30 μg), moxifloxacin (5 μg), ciprofloxacin (5 μg), and sulfamethoxazole-trimethoprim (1.25/23.75 μg) on Mueller–Hinton agar (Bio-Rad, Feldkirchen, Germany). The results were read using Adagio (Bio-Rad, Feldkirchen, Germany) and interpreted as sensitive, intermediate, or resistant according to the EUCAST breakpoints (version 2021) [18].

### 4.4. Quantitative Phenotypic Carbapenem Resistance

The minimum inhibitory concentration (MIC) of carbapenem antibiotics was determined using the E-test^®^ ertapenem strips in accordance with the manufacturer’s instructions (BioMérieux Deutschland GmbH, Nürtingen, Germany) for all meropenem-intermediate and -resistant *E. coli* isolates identified in the Kirby–Bauer disc diffusion method. According to the EUCAST 2021 breakpoints, the isolates were considered as resistant when the MIC breakpoint was greater than 0.5 g/mL [18].

### 4.5. DNA Extraction

*E. coli* strains were re-inoculated on blood agar (Oxoid, Cambridge, UK) and incubated aerobically for 18–22 h at 37 °C; 3–5 pure colonies were taken with an inoculating loop and suspended with nuclease-free water and extracted using a High Pure PCR template preparation kit (Roche, Mannheim, Germany) following the manufacturer’s instruction. The length, quantity, purity, and concentration of the extracted DNA was measured by a Nano Drop ND-100 (Thermo Fisher Scientific, Wilmington, NC, USA).

### 4.6. Characterization of ESBL- and Carbapenem-Resistant Isolates

All *E. coli* strains that exhibited resistance to cefotaxime, cefepime, cefoxitin, piperacillin-tazobactam, and meropenem in the phenotypic antimicrobial susceptibility tests were analyzed using the check-MDR CT103XL DNA microarray kits (Wageningen, Netherlands). This approach aimed to detect and identify genes associated with carbapenemase (IMP, VIM, KPC, NDM-1, and OXA-48), AmpC-type β-lactamase (AmpC), cefotaximase-Munich (CTX-M type), Temoneira β-lactamase (TEM), and sulfhydryl variant β-lactamase (SHV) using the DNA microarray technique. The manufacturer’s instructions were followed strictly to ensure the accuracy and quality of the data obtained.

### 4.7. Library Preparation and Multilocus Sequence Typing (MLST)

A field sequencing kit (SQK-LSK109 kit) was used to prepare the DNA library for nanopore sequencing. Following the manufacturer’s instruction, an individual barcode was added to the prepared DNA library using the rapid barcoding kit 96 (SQK-RBK110.96). Each barcoded DNA sample was pooled and then loaded into the SpotON flow cell on the Nanopore device. Finally, in accordance with the previous work by Ramakrishnan et al., 2022, MLST was carried out for seven housekeeping genes of *E. coli* (adk, fumC, icd, purA, gyrB, recA, and mdh) [37]. Thereafter, raw reads were base-called using ONT’s Guppy software v 6.0.

### 4.8. Bioinformatic Analysis

A fast K-mer-based tool, String MLST v0.6.3, was used for multilocus sequence typing to determine the sequence type for each isolate based on the Achtman scheme [38]. The base-called reads were then mapped to the concatenated sequences of the seven traditional housekeeping genes of *E. coli* strains using minimap2 v2.26-r1175 [39]. Alignments were also performed with Minimap2, and all aligned sequences in Sequence Alignment/Map (SAM) format were converted to Binary Alignment/Map (BAM), followed by sorting and indexing using SAMtools v1.19.2 to prepare for variant calling. Mpileup files were created using BCFtools v1.19, and variant calling was conducted with the same tool. Thereafter, the resulting Variant Call Format (VCF) files were normalized, filtered, and indexed for generating consensus sequences using BCFtools. The consensus sequences were then merged into a single multifasta file with in-house Python (version 3.11.4) script for multiple sequence alignment using MAFFT v7.526 [40]. This alignment was used as input for phylogenetic analysis in RAxML v8.2.12, with bootstrap set to 500 [41]. The best-scoring maximum likelihood tree was visualized and annotated using iTol v6.9.1 [42]. Additionally, the MLST allelic profiles were analyzed with the eBURST algorithm and visualized using Phyloviz v2.0 according to previous studies [43,44].

### 4.9. Quality Assurance

All the laboratory work was carried out strictly in line with the laboratory’s standard operating procedures. Using DensiCHEK plus (BioMérieux, Marcy-l’Étoile, France), the inoculum density of bacterial suspensions was standardized to 0.5 McFarland for all phenotypic susceptibility tests. The Mueller–Hinton agar plates (Bio-Rad, Feldkirchen, Germany) were evenly streaked and loaded with discs and E-test strips according to the EUCAST guidelines. Furthermore, the quality of the amplified DNA was checked by running on agarose gel and visualizing the input DNA fragment length distribution for each of the housekeeping gene PCR runs along with the positive control before proceeding to the library preparation for MLST and DNA microarray analysis.

### 4.10. Ethical Considerations

The study received approval from the Institutional Review Board (IRB) of Jimma University, Health Institute, under reference number IHRPG1/1087/21. It was also approved by the Ethics Committee of the Medical Faculty of Ludwig-Maximilians-Universität of Munich, Germany, with opinion number Project No: 21-0157. Written informed consent was obtained from patients and healthy participants prior to their recruitment in the previous studies from which these *E. coli* strains were obtained. In line with the consent form, culture and antibiotic susceptibility test results were promptly reported to the treating physician to ensure appropriate patient care management. Information related to microbes was used in this study, with no participant identifiers or clinical details included.

## 5. Conclusions

The high prevalence of CTX-M-15 and NDM-1 in multidrug-resistant *E. coli* isolates from both clinical and environmental samples underscores the increasing threat of AMR in Ethiopia. The identification of these resistant genes, along with the presence of internationally known high-risk clones in various niches, suggests potential routes of transmission and raises concerns about public health. This situation emphasizes the urgent need for a One Health approach, as it seems too late for barrier measures to isolate the strains in the hospital setting, as they have already escaped into the environment and the population. This study also shows the rapid change in resistance patterns, as within a few years NDM-1 has gone from not being detected in *E. coli* to being the most prevalent carbapenemase in *E. coli*. Human, animal, and environmental health strategies need to be integrated to address the AMR challenge in the study setting effectively. To mitigate the spread of these resistant strains, it is essential to strengthen antimicrobial stewardship programs and enhance infection prevention and control measures. Such actions are crucial for safeguarding public health and curbing the rise of AMR in Ethiopia.

## Figures and Tables

**Table 1:** Distribution of carbapenemase- and extended-spectrum beta-lactamase-encoding genes of *Escherichia coli* isolated from environmental, animal, and human samples in Jimma town.

Types of Antimicrobial Resistance Genes	Sources of *E. coli* Strains				Total
	Patients % (*n* = 226)	Healthy Humans % (*n* = 118)	Animals % (*n* = 159)	Environment % (*n* = 108)	% (*n* = 611)
**Carbapenemase-encoding genes**	**3.5 (8)**	**0**	**0.6 (1)**	**5.6 (6)**	**2.5 (15)**
NDM	2.2 (5)	0	0	5.6 (6)	**1.8 (11)**
OXA-48	0.9 (2)	0	0.6 (1)	0	**0.5 (3)**
OXA-48 + NDM	0.4 (1)	0	0	0	**0.2 (1)**
**ESBL-encoding genes**	**59.7 (135)**	**11.0 (13)**	**9.4 (15)**	**37.9 (41)**	**33.4 (204)**
CTX-M-15	55.3 (125)	9.3 (11)	7.5 (12)	30.6 (33)	**29.6 (181)**
CTX-M-9	1.3 (3)	0.9 (1)	1.3 (2)	2.7 (3)	**1.5 (9)**
CTX-M, ND	1.8 (4)	0	0.6 (1)	2.8 (3)	**1.3 (8)**
CTX-M-32	1.3 (3)	0	0	0	**0.5 (3)**
CTX-M-15 + 9	0	0.9 (1)	0	1.8 (2)	**0.5 (3)**
**AMPC-encoding genes**	**7.1 (16)**	**0**	**3.1 (5)**	**5.6 (6)**	**4.4 (27)**
CMY II (*n* = 11)	4.0 (9)	0	0.6 (1)	0.9 (1)	**1.8 (11)**
ACT/MIR (*n* = 10)	2.2 (5)	0	1.3 (2)	2.8 (3)	**1.6 (10)**
DHA (*n* = 5)	0.9 (2)	0	0.6 (1)	1.9 (2)	**0.8 (5)**
ACT/MIR + DHA (*n* = 1)	0	0	0.6 (1)	0	**0.2 (1)**
**TEM/SHV-encoding genes**	**41.2 (93)**	**5.1 (6)**	**8.2 (13)**	**34.3 (37)**	**24.4 (149)**
*TEM (WT)* (*n* = 144)	39.4 (89)	5.1 (6)	8.2 (13)	33.4 (36)	**23.6 (144)**
*SHV (WT) (n = 4)*	1.8 (4)	0	0	0	**0.6 (4)**
*TEM-104K* + 164C (*n* = 1)	0	0	0	0.9 (1)	**0.2 (1)**
**Total**	**66.8 (151)**	**11.9 (14)**	**12.6 (20)**	**54.6 (59)**	**39.9 (244)**

**Table 2:** The distribution of resistance genes in *E. coli* obtained from patients and other sources.

Types of Antimicrobial Resistance Genes	Sources of the Resistant Strains		Total
	Patients (*n* = 151)	Others (*n* = 93)	% (*n* = 244)
**Co-existing in carbapenemase-producing strains**	**5.3 (8)**	**7.5 (7)**	**6.2 (15)**
NDM + CTX-M-15 + TEM	1.3 (2)	2.2 (2)	**1.6 (4)**
NDM + CTX-M-15	0.66 (1)	3.2 (3)	**1.6 (4)**
OXA-48 + CTX-M-15 + TEM	1.3 (2)	1.1 (1)	**1.2 (3)**
NDM + CMY II + CTX-M-15 + TEM	0.66 (1)	0	**0.4 (1)**
OXA-48 + NDM + CTX-M-15 + TEM	0.66 (1)	0	**0.4 (1)**
NDM + CTX-M, ND + TEM	0	1.1 (1)	**0.4 (1)**
NDM	0.66 (1)	0	**0.4 (1)**
**ESBL-producing strains**	**85.4 (129)**	**65.6 (61)**	**77.9 (190)**
CTX-M-15 + TEM	39.1 (59)	28.0 (26)	**34.8 (85)**
CTX-M-15	31.1 (47)	20.4 (19)	**27.1 (66)**
CTX-M-9	2.0 (3)	5.4 (5)	**3.3 (8)**
CTX-M-15 + CMY II + TEM	2.6 (4)	0	**1.6 (4)**
CTX-M-15 + CMY II	1.3 (2)	2.2 (2)	**1.6 (4)**
CTX-M group 1, ND + TEM	2.0 (3)	0	**1.2 (3)**
CTX-M-15 + TEM + SHV	2.0 (3)	0	**1.2 (3)**
CTX-M-32 + TEM	2.0 (3)	0	**1.2 (3)**
CTX-M group 1, ND	0.66 (1)	2.2 (2)	**1.2 (3)**
CTX-M-15 + 9 + *TEM*	0	2.2 (2)	**0.8 (2)**
CTX-M-15 + ACT/MIR	1.3 (2)	0	**0.8 (2)**
CTX-M-15 + SHV	0.66 (1)	0	**0.4 (1)**
CTX-M-15 + DHA	0.66 (1)	0	**0.4 (1)**
CTX-M group 1, ND + ACT/MIR + DHA	0	1.1 (1)	**0.4 (1)**
CTX-M-15 + ACT/MIR + TEM	0	1.1 (1)	**0.4 (1)**
CTX-M-9 + ACT/MIR + *TEM*	0	1.1 (1)	**0.4 (1)**
CTX-M-15 + 9	0	1.1 (1)	**0.4 (1)**
CTX-M-15 + DHA + *TEM-104K* + 164C	0	1.1 (1)	**0.4 (1)**
**AMPC-encoding genes**	**4.0 (6)**	**5.4 (5)**	**4.5 (11)**
ACT/MIR	2.0 (3)	3.2 (3)	**2.4 (6)**
CMY II + TEM	1.3 (2)	0	**0.8 (2)**
DHA	0	2.2 (2)	**0.8 (2)**
DHA + TEM	0.66 (1)	0	**0.4 (1)**
**TEM-encoding genes**	**5.3 (8)**	**21.5 (20)**	**11.5 (28)**
*TEM- (WT)*	5.3 (8)	21.5 (20)	**11.5 (28)**

## Data Availability

The data will be available from the corresponding author upon reasonable request.
